# Prevalence of Childhood Obesity Among Children and Adolescents in Saudi Arabia: A Systematic Review

**DOI:** 10.7759/cureus.70135

**Published:** 2024-09-24

**Authors:** Tasneem R Adam, Ahmed M Hamed, Heba Saad M Mohammed, Tarteel Elryahi Elsayed Elshareef, Hanan Mushaeb, Awad Nafel A Al Harbi, Boran M Bawarith, Ahmed Abdullah Almalki, Nawal Alzaheb, Abdulaziz Hassan Alqarni, Mona Abdelbaky

**Affiliations:** 1 Dental Health, College of Applied Medical Sciences, King Saud University, Riyadh, SAU; 2 Department of Stroke, United Lincolnshire Hospital Trust, Lincolnshire, GBR; 3 Faculty of Medicine and Surgery, University of Malta, Malta, MLT; 4 Emergency Medicine Department, Rwad Elshefa Medical Complex, Muhayil Aseer, SAU; 5 Medical School, King Abdulaziz University, Faculty of Medicine, Jeddah, SAU; 6 Family Medicine Department, Medical Center in the Security Forces Facilities, Riyadh, SAU; 7 Anesthesia Department, King Abdullah Medical Complex, Jeddah, SAU; 8 Emergency Medicine, Saudi Red Crescent Authority, Riyadh, SAU; 9 Epidemiology and Public Health, Ministry of Health, Riyadh, SAU; 10 General Practice, Khamis Mushayt General Hospital, Abha, SAU; 11 Neonatology, Prince Sultan Military Medical City, Riyadh, SAU

**Keywords:** childhood obesity, epidemic, prevalence, public health, saudi arabia

## Abstract

Childhood obesity is a growing public health concern worldwide, with significant implications for long-term health outcomes. Thus, the aim of this study is to highlight the prevalence and trend of obesity among children and adolescents in Saudi Arabia over the last 24 years. This systematic review included participants aged 2 to 19 years without systemic disease, reporting the prevalence of obesity using the Centers for Disease Control and Prevention (CDC) classification and BMI calculation, from studies in English or Arabic published between January 2000 and April 2024. We conducted a comprehensive search across multiple databases including ProQuest, Google Scholar, ISI Web of Science, Embase via Ovid, and MEDLINE via Ovid, and reviewed references of included studies. Data were extracted and quality assessed independently by two authors, with any disagreements resolved through discussion with a third reviewer, using the Newcastle-Ottawa Scale modified for this study. This systematic review included 21 studies from Saudi Arabia, published between 2006 and 2023, with participants aged 2 to 19 years. The studies involved a total of 63,512 subjects. Among children, the prevalence of overweight ranged from 5% to 29%, while obesity ranged from 3.8% to 49.7%, classified using CDC criteria. Quality assessment rated 10 studies as Very Good, 10 as Good, and one as Unsatisfactory. The systematic review of childhood obesity prevalence in Saudi Arabia over the past 24 years highlights alarming trends and significant public health implications. Our analysis emphasizes an increase in obesity rates among children and adolescents, revealing a complex link of socioeconomic, cultural, and lifestyle factors contributing to this epidemic.

## Introduction and background

In the 1970s, obesity among children and adolescents was a relatively rare condition; however, nowadays, obesity has become more common than ever. The prevalence of childhood obesity has progressively increased over the last few decades, affecting more than 330 million children and adolescents [1]. Childhood obesity has seen a significant rise, increasing from a mere 4% in 1975 to slightly over 18% in 2016 [1,2]. In 2019, the World Health Organization (WHO) reported that about 38.2 million children under the age of 5 years were affected by overweight or obesity [2].

Childhood obesity poses significant risks to both short-term and long-term health outcomes for children. Excess weight can lead to various health issues, including high cholesterol levels, type 2 diabetes, respiratory difficulties, joint problems, and gallstones [3]. Childhood obesity can also contribute to decreased self-esteem, depression, anxiety, and social challenges, such as bullying and stigmatization [4]. Furthermore, obese children are more likely to become obese adults, which increases their risk of developing conditions like stroke, various cancers, premature mortality, and mental health disorders, such as clinical depression and anxiety [4,5]. Additionally, childhood obesity significantly impairs the overall quality of life for affected children, impacting their physical, emotional, and social well-being [6].

Saudi Arabia has witnessed remarkable advancements in treating various childhood health conditions over the last 30 years. However, childhood obesity stands out as a “new morbidity,” with its prevalence reaching unique levels. A national survey conducted from 2020 to 2022 among 1,120,417 children and adolescents across Saudi Arabia revealed that the prevalence of overweight and obesity was 116,547 (10.4%) and 119,610 (10.7%), respectively [7]. Moreover, a recent study in Riyadh, Saudi Arabia, raised substantial health concerns among children, reporting an overall prevalence of obesity was 18.2% [8]. In 2022, another study in the Eastern Province of Saudi Arabia conducted a study among 20,000 Saudi children found even more alarming figures, with overweight and obesity levels among high school children reaching 25.7% [9].

A thorough understanding of the childhood obesity epidemic and its risk factors is essential for guiding intervention strategies and the development of effective population-based programs and policies. Thus, this systematic review aims to highlight the prevalence and trend of obesity among children and adolescents in Saudi Arabia over the last 24 years.

## Review

Method

Eligibility Criteria

The inclusion criteria were as follows: (a) participants between the ages of 2 and 19 years who are generally healthy without systemic disease; (b) studies reporting the prevalence of obesity using the CDC obesity classification, with BMI calculated as weight in kg/(height in m²); (c) studies in English or Arabic published between January 2000 and April 2024; and (d) population-based studies conducted in Saudi Arabia. We excluded all papers that did not completely comply with the inclusion criteria, as well as abstracts without full texts, conference papers, and studies with self-reported obesity.

Search Strategy

The following databases were thoroughly searched for studies that met the inclusion criteria: ProQuest, Google Scholar, ISI Web of Science, Embase via Ovid, and MEDLINE via Ovid. Refer to Appendix A for detailed information about each database’s search strategy. TA and SN reviewed the references of included studies to find relevant papers. The citations that have been identified through the search are imported into Endnote X8.

Data Extraction and Assessment of Study Quality

An Excel sheet with a standardized format was utilized to extract relevant information. This format underwent a pilot test with five selected studies included in the review. Two authors independently extracted the data from the selected studies. Any disagreements in the extracted and assessed data were resolved through discussion with a third reviewer. Information extracted included: the author’s name, year of examination, age of participants, study design, gender, location, sample size, measurement methods, and prevalence of obesity. The quality of the included studies was evaluated independently by two authors using the Newcastle-Ottawa Scale for cross-sectional investigations [10], modified for this study [11,12]. The scale assessed three domains: selection, comparability, and outcome. Each article was categorized as Very Good Studies (9-10 points), Good Studies (7-8 points), Satisfactory Studies (5-6 points), and Unsatisfactory Studies (0-4 points).

Results

**Figure 1:**
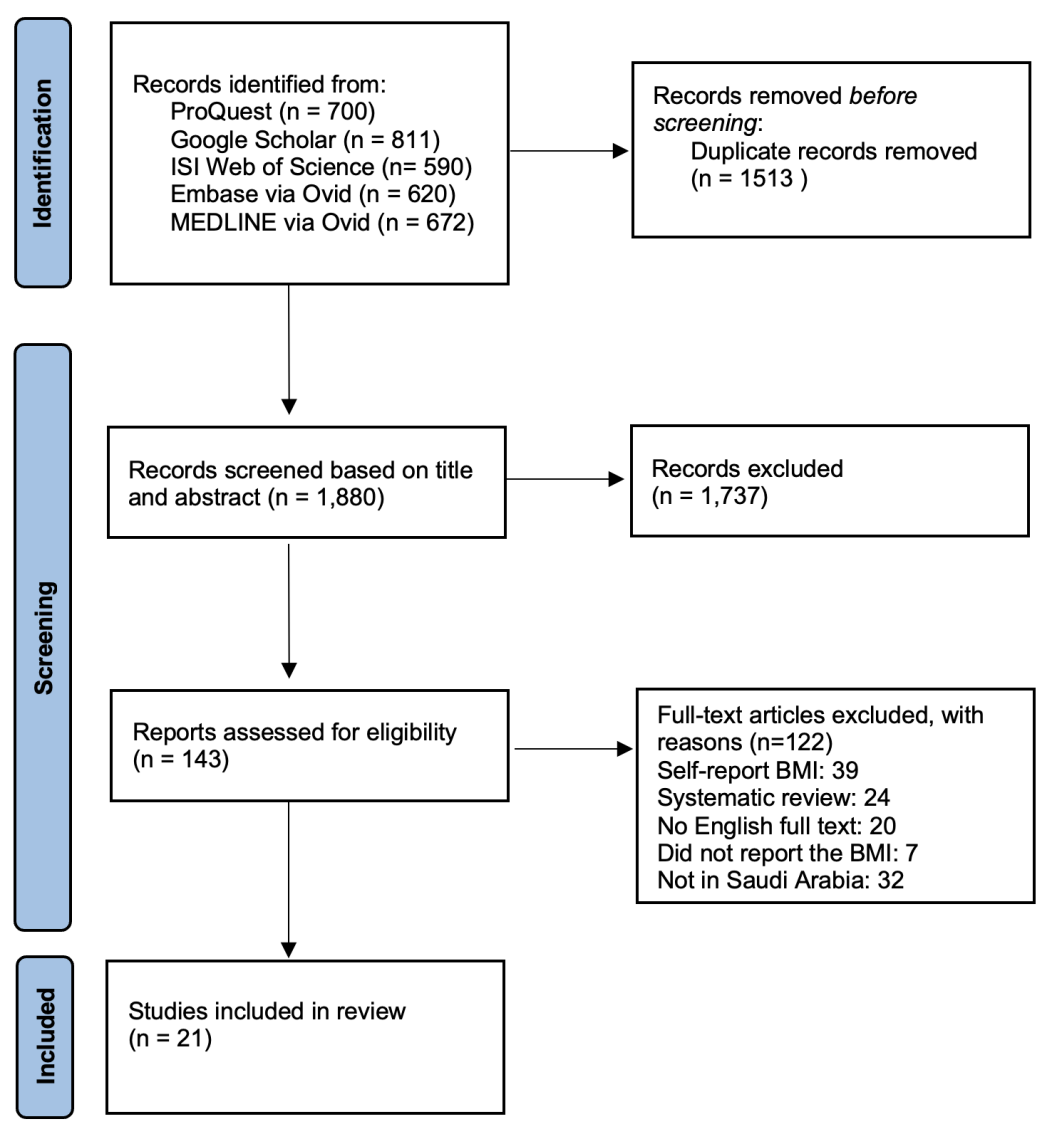
The flow diagram detailing the study selection process for the systematic review of childhood obesity studies in Saudi Arabia The flow diagram was created by the authors of this article.

The search retrieved 3,393 papers from January 1945 to April 2024. Of these, 1,513 were duplicates and excluded. After reviewing titles and abstracts, 1,737 papers were deemed irrelevant due to geographical irrelevance, language restrictions, or being duplicate publications. As a result, 143 papers were considered for full-text screening, of which 21 articles met all inclusion criteria. The study selection process is illustrated in Figure 1 using the Preferred Reporting Items for Systematic Reviews and Meta-Analyses (PRISMA) flowchart.

All studies employed cross-sectional designs. The included papers were published between 2006 and 2023, covering data collected from 2003 to 2020. The total number of participants ranged from 102 to 19,317 across 63,512 subjects in the included studies. Five studies reported childhood obesity as a secondary outcome [13-17]. All studies utilized calibrated scales for weighing children, while 10 studies trained their teams for data collection to ensure internal calibration [14-16,18-24]. Moreover, all studies classified obesity using the CDC criteria. Two studies were conducted nationwide in Saudi Arabia [25,26], while five studies focused on Riyadh. Additionally, there were two studies each in Al-Khobar, Jeddah, and one study in Tabuk. Individual studies were conducted in Al-Hasa, Al-Kharj, Aljouf (including Sakaka, Qurayyat, and Dumat Al-Jandal), Arar, Jazan, and Makkah. One study covered three regions: Central, Southwest, and North. Eight studies included only female students, four included only male participants, eight included both males and females, and one study did not specify the gender of the subjects [14]. The average age of participants ranged from 2 to 19 years, with only one study including a participant who was 20 years old. However, the mean age was 16.8 years [27]. The prevalence of overweight ranged from 5% to 29%, while obesity ranged from 3.8% to 49.7%. One study reported the combined prevalence of overweight and obesity. Table 1 summarizes the key characteristics of the selected studies.

**Table 1:** Characteristics of Included Studies

Study ID	Data Collection Period	Sample Size	Gender	Age	Location	Prevalence of Overweight BMI: 85th - 95th Percentiles	Prevalence of Obesity BMI > 95th Percentile
Al-Saeed et al. 2007 [18]	Jan to Mar 2003	2239	Female	6-17	Al-Khobar	20.00%	11.30%
Al-Rowaily et al. 2007 [19]	2003-2005	6207	Both	4-8	Riyadh	325 (5.4%)	243 (4%)
Collison et al. 2010 [20]	2007 academic year	9433	Both	10-19	Riyadh	12.2%	27.00%
El Mouzan et al. 2010 [25]	Data from 2005 growth charts	19,317	Both	5-18	All SA	17 825 (20.4%)	18 625 (5.7%)
Abahussain 2011 [28]	2007	321	Female	15-19	Al-Khobar	30% Overweight and obesity combined	
El Mouzan et al. 2012 [21]	2004 and 2005	11,112	Both	2-17	Central, Southwest, North	2,037 (18.3%)	913 (8.2%)
Alenazi et al. 2015 [29]	March and April 2012	523	Male	15-19	Arar	17.20%	30.40%
Hothan et al. 2016 [30]	Mar 2015 Jun 2015	401	Both	11-18	Jeddah	60 (15%)	77 (19.2%)
Saleh et al. 2017 [23]	Jan 2016 -May 2016	240	Male	7-15	Al Hasa	26 (10.8%)	9 (3.8%)
Al-Kutbe et al. 2017 [22]	January to May 2014	266	Female	8-11	Makkah	34 (12.8%)	46 (17.3%)
Quadri et al. 2017 [13]	Not mentioned	360	Both	6-15	Jazan	23 (6.4%)	17 (4.7%)
Abu El Qomsan, et al. 2017 [14]	Oct 2014 to May 2015	386	Not mentioned	6-12	Al-Kharj city	71 (18.39%)	192 (49.74%)
Nasreddine et al. 2018 [26]	2011-2012	7278	Both	10-19	All SA	Not reported	1176 (16.16%)
Alturki et al. 2018 [24]	Dec 2015 to Mar 2016	1023	Both	9-12	Riyadh	Not reported	497 (48.5%)
Almuhlafi et al. 2018 [27]	Academic year 2017-2018	399	Female	2-20	Tabuk	65 (16.3%)	
Bandy et al 2019 [17]	Jan 2018 and Mar 2018	400	Male	15-17	Aljouf	80 (20%)	134 (33.5%)
Abdellatif et al. 2020 [15]	Not mentioned	2247	Female	12-15	Riyadh	226 (10.1%)	112 (5.0%)
Alshumrani et al 2020 [31]	2017 and 2018	681	Female	Grade one to six	Jeddah	18.70%	17.80%
Alhusaini et al. 2020 [32]	Aug 2015 to Aug 2016	275	Female	10-16	Riyadh	19.60%	18.20%
Gudipaneni et al. 2021 [16]	Dec 2018 and March 2019	302	Male	12-14	Aljouf province (Sakaka, Qurayyat, and Dumat Al-Jandal)	54 (17.9%)	40 (13.2%)
Gad et al. 2023 [33]	2019-2020	102	Female	12-18	Tabuk	29.40%	8.80%

All the included studies were based on a cross-sectional design, with three studies utilizing national datasets. Each study used a validated screening tool, specifically the 2000 Center for Disease Control (CDC) growth reference. Nearly one-third of the studies (7 studies, 33%) employed a multistage sampling technique. Four studies did not describe the sampling method, and one study used a convenience sample. In terms of quality assessment, 10 studies were categorized as Very Good Studies, scoring between 9 and 10 points. Another 10 studies were classified as Good Studies, with scores ranging from 7 to 8 points. Only one study was deemed Unsatisfactory, with a score between 0 and 4 points (Table 2).

**Table 2:** Newcastle-Ottawa Scale (NOS) for Assessing the Quality of Cross-Sectional Studies Two stars (★★) are awarded only for the “Ascertainment of the screening/surveillance tool” and “Assessment of the outcome” criterion.

	Selection				Comparability	Outcome			
	Representativeness of the sample	Sample size justified and satisfactory	Non-response rate	Ascertainment of the screening/surveillance tool	Comparability of cohorts	Assessment of the outcome		Statistical test	Total
Study ID					Main factor	Additional factor	Scale		
Al-Saeed et al. 2007 [18]	★	★	0	★★	★	★	★★	★	9/10
Al-Rowaily et al. 2007 [19]	★	★	★	★★	★	0	★★	★	9/10
Collison et al. 2010 [20]	★	★	★	★★	★	0	★	0	7/10
El Mouzan et al. 2010 [25]	★	★	★	★★	★	★	★★	★	10/10
Abahussain 2011 [28]	★	★	★	★★	★	★	★	★	10/10
El Mouzan et al. 2012 [21]	★	★	★	★★	★	0	★★	0	8/10
Alenazi et al. 2015 [29]	★	★	★	★★	★	★	★	★	10/10
Hothan et al. 2016 [30]	★	★	★	★★	★	★	0	★	8/10
Saleh et al. 2017 [23]	★	★	0	★★	★	★	★★	★	9/10
Al-Kutbe et al. 2017 [22]	★	★	0	★★	0	0	★★	★	7/10
Quadri et al. 2017 [13]	★	★	★	★ ★	0	★	★	★	8/10
Abu El Qomsan et al. 2017 [14]	★	★	0	★ ★	★	★	★★	★	9/10
Nasreddine et al. 2018 [26]	★	★	0	★ ★	★	★	★	★	8/10
Alturki et al. 2018 [24]	★	★	0	★ ★	★	★	★★	★	9/10
Almuhlafi et al. 2018 [27]	★	★	0	★ ★	★	★	★	★	8/10
Bandy et al. 2019 [17]	★	★	★	★ ★	★	★	★	★	9/10
Abdellatif et al. 2020 [15]	★	★	★	★ ★	★	0	★★	★	9/10
Alshumrani et al 2020 [31]	0	★	0	★ ★	★	★	★	★	7/10
Alhusaini et al. 2020 [32]	0	★	0	★ ★	★	★	★	★	7/10
Gudipaneni et al. 2021 [16]	★	★	0	★ ★	0	0	★★	★	7/10
Gad et al. 2023 [33]	0	0	0	★ ★	0	0	★	★	4/10

**Figure 2:**
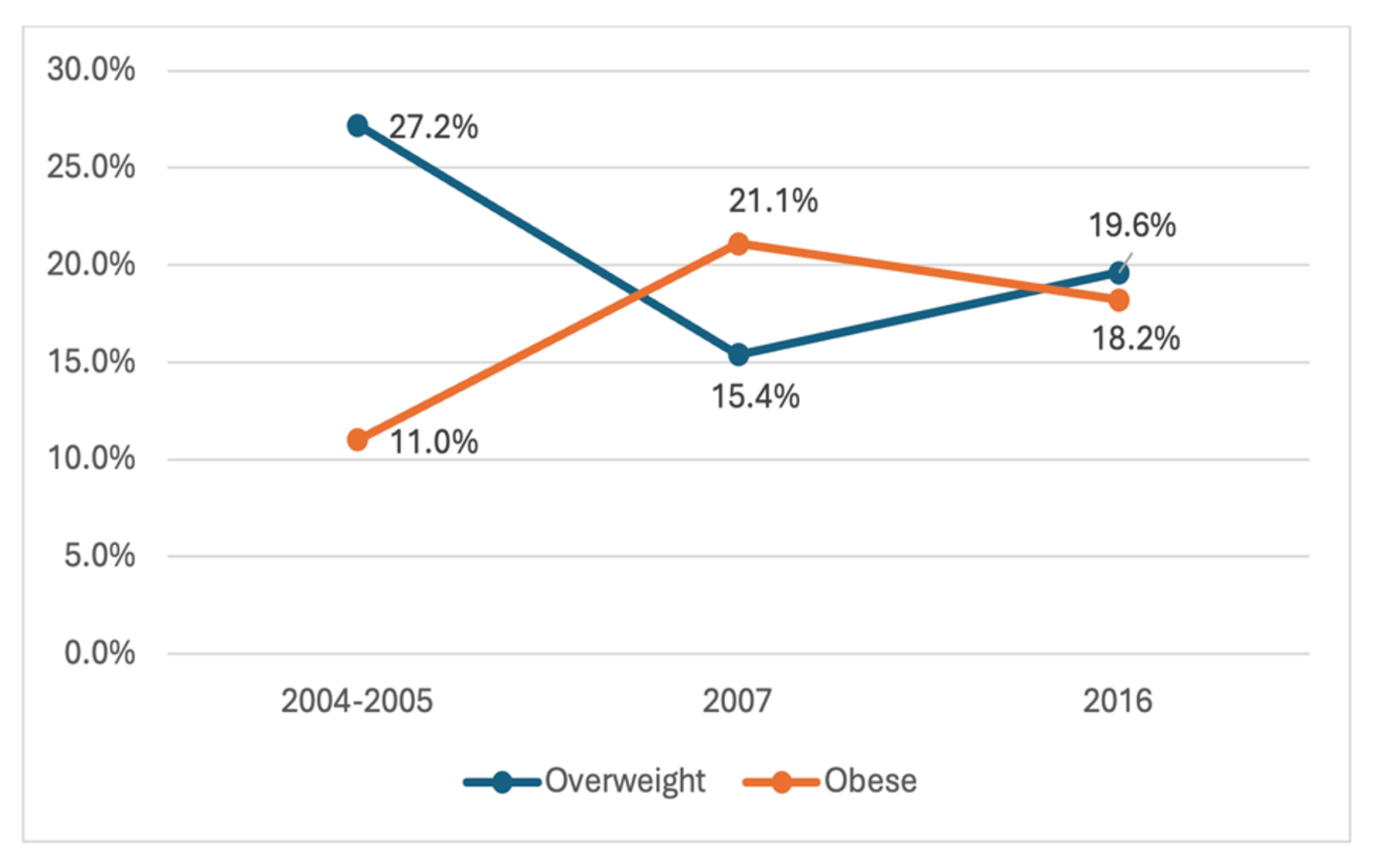
Trend of prevalence of obese children aged 2-12 (Riyadh) The image was created by the authors of this article.

The line chart in Figure 2 shows the trend of prevalence of overweight and obese adolescents aged 13-19 in Riyadh for the years 2004-2005, 2007, and 2016. In 2004-2005, 27.2% were overweight and 11.0% were obese. By 2007, overweight rates fell to 15.4%, while obesity nearly doubled to 21.1%. In 2016, overweight rates rose slightly to 19.6%, and obesity decreased to 18.2%. These trends indicate shifting patterns in adolescent overweight and obesity rates in Riyadh.

**Figure 3:**
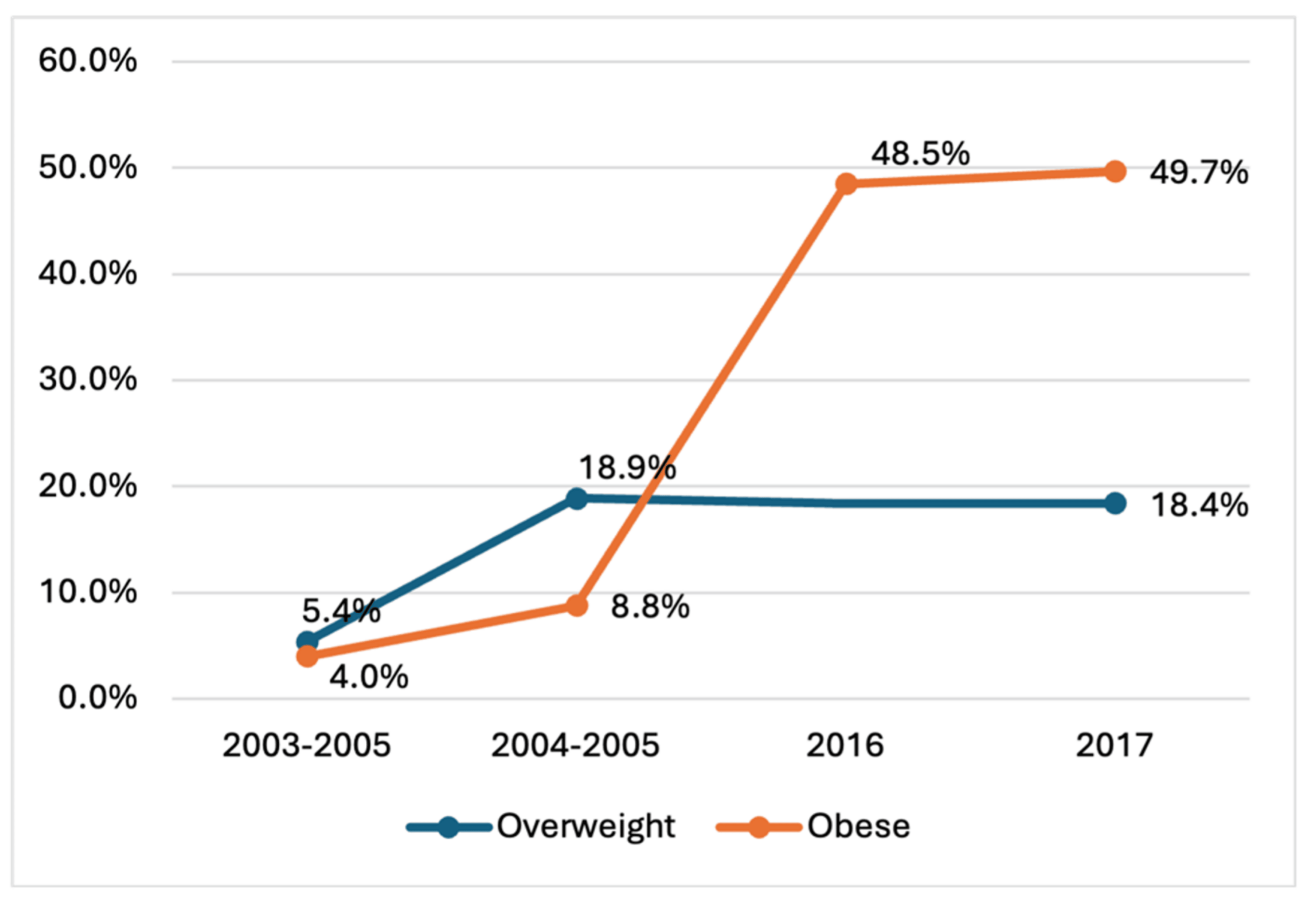
Trend of prevalence of overweight and obese adolescents aged 13-19 (Riyadh) The image was created by the authors of this article.

Four studies were conducted in the Riyadh region, and one in the Central region (covering Qassim and Riyadh). We were able to provide an overview of the trend in the prevalence of obesity and overweight among school-age children. Figure 3 illustrates the trend of prevalence of obese children aged 2-12 in Riyadh across four different periods: 2003-2005, 2004-2005, 2016, and 2017. The prevalence of obesity among these children has significantly increased over time. Starting at 4.00% in 2003-2005, the rate more than doubled to 8.77% in 2004-2005 and then surged to 48.50% in 2016. By 2017, the prevalence of obesity had slightly increased to 49.70%. This data highlights a concerning rise in obesity rates among children in Riyadh (Figure 2).

**Figure 4:**
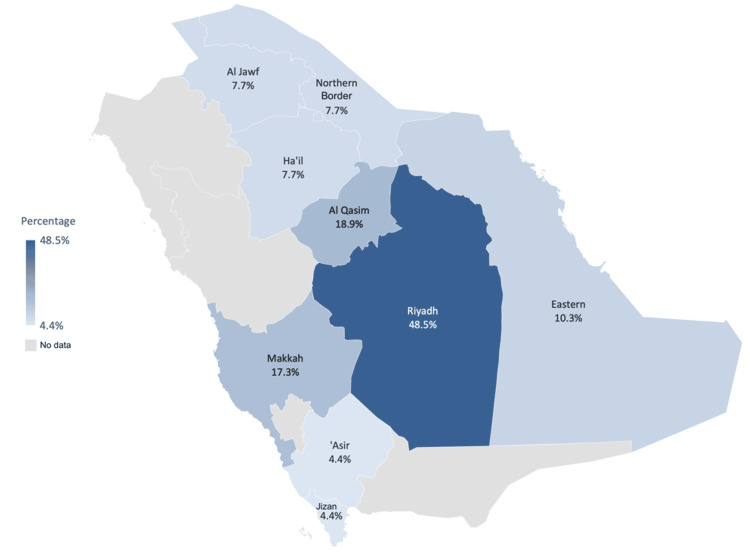
Map showing the prevalence of obesity in children aged 2-12 by region Based on the most recent published data range between 2003 and 2016. The image was created by the authors of this article.

Figure 4 illustrates the prevalence of obesity among children aged 2-12 in Saudi Arabia by region. The Riyadh region has the highest rate at 48.5%, followed by the Makkah region at 17.3% and the Qassim region at 18.9%. The North regions (Hail, Al Jawf, and Northern Borders) uniformly report 7.7%, while the Southwest region (Asir and Jizan) has the lowest rate at 4.4%. The Eastern province (Al-Khubar) shows a moderate rate of 10.3%.

**Figure 5:**
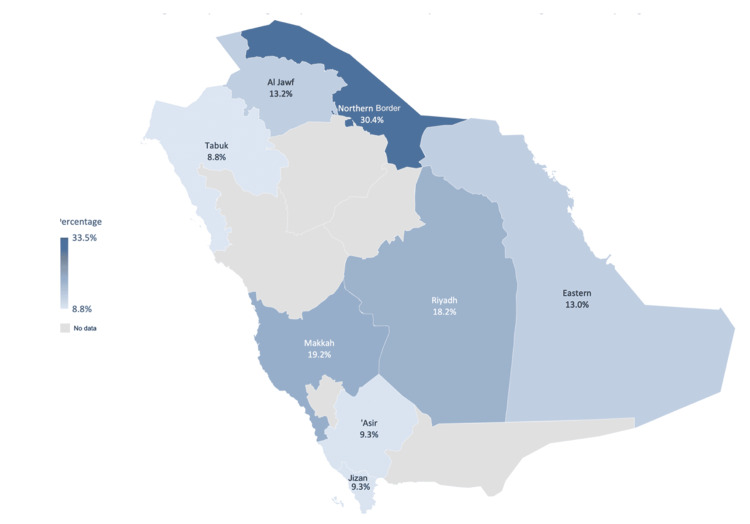
Map showing the prevalence of obesity in adolescents aged 13-19 Based on the most recent published data range between 2004 and 2020. The image was created by the authors of this article.

Figure 5 shows obesity prevalence among adolescents aged 13-19 in Saudi Arabia by region. The Northern Border has the highest rate at 30.4%, followed by the Makkah region at 19.2% and the Riyadh region at 18.2%. The Tabuk shows the lowest rate at 8.8%. Other regions include 13.2% in the Al Jowf, 13.0% in the Al-Khubar, and 9.3% in the Asir and Jizan regions, indicating varied obesity rates across the country.

Discussion

This systematic review examines published studies on the prevalence of childhood obesity in the Kingdom of Saudi Arabia (KSA) over the last two decades. To our knowledge, this is the first systematic review to assess the prevalence and trends in childhood obesity in KSA using the CDC obesity classification, making our review more accurate and providing a comprehensive overview of the rates of childhood obesity. Our review reveals a significant increase in the prevalence of obesity among children aged 6-12 in the Riyadh region. Specifically, the prevalence of obesity in the Riyadh region was found to be 4% in 2003-2004 [19], compared to 48.5% in 2016 [24] for both males and females aged between 4 and 12. Additionally, the rate of overweight among children increased from 5.4% [19] to 18.3% [14] in the Riyadh region. Unfortunately, other regions, such as the Makkah region, also witnessed a significant increase in overweight and obese children [22,31].

This study illustrates the diversity in the prevalence of overweight and obesity among different regions in Saudi Arabia. For instance, the prevalence of obese children in the Riyadh region was significantly higher than in the Makkah region, at 48.5% and 17.3%, respectively. Moreover, the prevalence of obesity among adolescents was found to be 33.5% in Sakaka, compared to 13.2% in the Aljouf province during the same period among males. Overweight among children was nearly the same in Al-Kharj city and Jeddah in 2017, at 18.39% and 18.70%, respectively. Among adolescents, the rate of overweight was 20% in Sakaka, while it was 17.9% in the Aljouf province among male subjects.

This global crisis affects children in both developed and underdeveloped nations, making it a top-priority public health issue worldwide [34]. In Saudi Arabia, the connection between family income and childhood obesity follows a unique pattern compared to what’s typically observed in other places. A study involving schoolchildren aged 12-19 in the Hail region reported an unexpected result. It showed that families with a monthly income of less than 5000 Saudi Riyals had a lower likelihood of having obese children [35]. Another study conducted in Jeddah, among school-age children, the study found that 47.4% of obese children came from high-income families [36]. A 2023 study examining the impact of socioeconomic factors on obesity among adolescent students in Asser found that family income is a significant predictor of weight status, with higher family income being associated with an increased likelihood of being overweight or obese [37]. Among the included studies, only two investigated the impact of socioeconomic factors on childhood obesity. Al-Saeed et al. did not find an association between socioeconomic status and obesity [18]. Hothan et al. discussed various findings related to the health and lifestyle of the children involved, including the prevalence of hypertension, obesity, and other health markers. However, this study did not report the impact of family income on its results [30]. Thus, while global trends highlight the widespread impact of socioeconomic status on children's health [38-40], studies from Saudi Arabia reveal a unique relationship between family income and childhood obesity. This finding suggests that as family income increases, there is a higher prevalence of child obesity within the confines of Saudi Arabia.

During the preschool years, children form their dietary habits. Parents play a major role in establishing these habits during this critical period [41,42]. By the time children reach adolescence, their understanding of the long-term effects of their habits is often limited. A 2017 study conducted in Al-Hasa investigated the impact of working parents on childhood obesity among 240 children aged 7-15 years [23]. The study found that the employment status of fathers was not significantly associated with the child's BMI. In contrast, the employment status of mothers showed a strong and statistically significant association with the child's BMI (p-value < 0.0001). Children of working mothers had a higher prevalence of overweight and obesity. This could be due to factors such as increased consumption of pre-packaged foods, eating out, and substituting main meals with fast food. Furthermore, both fathers’ and mothers’ obesity were significantly associated with childhood obesity. Furthermore, parental characteristics such as BMI, eating behaviors, and awareness of the extent of the obesity burden also play a pivotal role in shaping the risk of obesity among Saudi children [43,44]. It is quite unusual to observe overweight children in families where the parents are slim.

Nowadays, it has become common knowledge that fast food is cheap, tasty, more convenient, heavily marketed, calorie-dense, and widely available food, but if ungoverned it becomes a liability to the health of the population. The food industry plays a major role in child obesity [45,46]. While some may argue that parents are always responsible and have a duty to ensure their child’s health, other segments, such as society and the food industry, share a larger responsibility. A 2020 study demonstrated the extreme measures the food industry will take the extra step to keep the public unaware of the reality of the food they are buying. The study indicated that the food and beverage industry actively shapes food environments and influences nutrition policies through various strategies, including lobbying governments, questioning opposing scientific evidence, funding research supporting their position, emphasizing personal responsibility for obesity, and employing numerous other tactics [47]. As a result, the industry's influence has been identified as a barrier to nutrition policy change. When parents lack knowledge about what makes a balanced and nutritious diet or lack the time to cook nutritious meals for their children, the kids are essentially left to make food choices on their own. Meanwhile, the food industry knowingly targets and exploits these young consumers by prominently displaying unhealthy food in most grocery stores, especially in ways that attract children's attention. The fight against childhood obesity is an uneven struggle, where a single child must contend with billion-dollar industries that employ various tactics to persuade the child and even their parents to purchase their products.

In Saudi Arabia, cultural and dietary factors significantly contribute to childhood obesity. Studies have linked childhood obesity to dietary habits such as the consumption of sweets, chocolate, and fast food [43,48,49]. Nasreddine et al. found that nearly 60% of the 7278 participants did not consume fruit daily, and an equal percentage reported not drinking milk at all. Moreover, 60% of the participants indicated they drank two or more soft drinks each day [26]. Similarly, Alturki et al. found a high frequency of fast-food consumption (weekly intakes) being much higher in obese children [24]. Furthermore, there is a correlation between soft drink consumption and BMI in boys aged 10-19. Soft drink consumption was also linked to unhealthy eating habits in both males and females, such as consuming ice cream desserts, savory snacks, and fast-food meals. Conversely, for both genders, the consumption of milk was positively connected with the intake of fruits, vegetables, eggs, cheese, and dates, and negatively correlated with BMI and waist circumference [20]. A systematic review investigating the social determinants of overweight and obesity among adolescents in KSA found that rapid economic growth in the Kingdom has led to significant dietary changes among adolescents. This includes increased consumption of unhealthy foods and beverages, which are statistically associated with higher rates of overweight and obesity [22].

Insufficient physical activity and sedentary behaviors significantly contribute to the issue of childhood obesity in Saudi Arabia [50,51]. Research has shed light on the low levels of physical activity among Saudi children and adolescents, who frequently engage in sedentary activities such as watching TV, playing video games, and using electronic devices [51]. In a study by Al-Kutbe et al., it was found that female students in Saudi Arabia ages 8 to 11, exhibited very low levels of physical activity across all BMI groups. None of the participants achieved the daily recommended 60 minutes of moderate-to-intense activity, and less than 10% reached the daily goal of 10,000-12,000 steps [1]. Contributing factors to this problem include a heavy dependence on cars for short-distance travel, even for school commutes, and the inadequacy of comprehensive physical education programs, especially for girls [43].

The included studies exhibit several strengths that bolster the robustness of the findings. Many studies utilized validated screening tools, such as the CDC growth reference, ensuring consistency in the measurement of obesity across different studies. Additionally, some studies employed national datasets, providing a comprehensive overview of the prevalence of childhood obesity across various regions in Saudi Arabia. This use of large, representative samples enhances the generalizability of the results. However, the review also identified some limitations within the included studies. A significant proportion of the studies did not measure the relationship between risk factors and obesity.

Intervention is urgently needed due to the significant increase in the prevalence of overweight and obesity among children and adolescents in KSA. It is essential to incorporate daily physical activity, food, and nutrition into the school curriculum and to educate children, adolescents, and families about the negative health effects of obesity, such as diabetes and heart disease. Schools are the best places to ensure that these interventions are carried out successfully and to make health promotion more accessible.

Furthermore, it is crucial to adopt and implement national regulations that restrict the food industry from marketing ultra-processed foods high in sodium, trans fats, or sugar in their advertisements by using the term “healthy” or any comparable phrase. This measure is essential to avoid misleading the population.

Childhood obesity is a multifaceted problem, that requires efforts from individuals, corporations, and governments [52]. Additionally, different factors affect children differently, highlighting the need for a nuanced understanding of how obesity prevention and intervention programs operate. Realist reviews and evaluations serve as essential tools in this regard. These approaches enable a thorough exploration of the underlying causes and the specific conditions in which childhood obesity occurs. By focusing not only on whether a program is effective or not but also on why, how, and for whom. Realist reviews and evaluations allow for program optimization and adaptation to local contexts and different target populations. Moreover, more research is needed to explore the genetic and biological factors contributing to childhood obesity in the Saudi population.

## Conclusions

This systematic review of childhood obesity prevalence in Saudi Arabia over the past 24 years reveals alarming trends with significant public health implications. The analysis shows a concerning rise in obesity rates among children and adolescents, driven by a complex interplay of socioeconomic, cultural, and lifestyle factors. The findings highlight the urgent need for targeted interventions, such as early promotion of healthy lifestyles, enhanced nutritional literacy, and school-based physical activity and nutrition education. Additionally, regulations limiting the marketing of unhealthy foods to children are crucial for mitigating the impact of the food industry.

Despite the introduction of policies targeting obesity, such as increasing sugary drink prices and requiring food industries to disclose calorie counts, no studies have evaluated their impact on childhood obesity. This underscores the need for further research, including investigations into genetic and biological factors. Addressing childhood obesity in Saudi Arabia demands collaborative efforts from healthcare providers, policymakers, educators, and families to create a supportive environment and implement evidence-based interventions, ensuring healthier futures for the nations children and adolescents.

## Appendices

Appendix A (Search strategy)

*MEDLINE via Ovid* 

#1 (Saudi Arabia OR Saudi Arabia OR Saudi OR Arabia OR Riyadh OR Mecca OR Medina OR Jeddah OR Makkah).ti,ab,kf.

#2 exp Saudi Arabia/

#3 #1 OR #2

#4 (Pediatric Obesity OR Obesity OR Overweight OR BMI OR Body Mass Index OR Adiposity OR Obesity related diseases OR obesities).ti,ab,kf.

#5 exp Pediatric Obesity/

#6 #4 OR #5

#7 (Child OR Adolescent OR Child* OR children* OR Childhood OR Kid OR kids OR Minor OR Toddler OR Teenager* OR Young adult* OR adolescent OR adolescence OR adolescences).ti,ab,kf.

#8 exp Child/ OR exp Adolescent/

#9 #7 OR #8

#10 (Prevalence OR Epidemiology OR Cross-Sectional Studies OR epidemiology).ti,ab,kf.

#11 exp Prevalence/ OR exp Epidemiology/ OR exp Cross-Sectional Studies/ OR epidemiology.fs.

#12 #10 OR #11

#13 #3 AND #6 AND #9 AND #12

Embase via Ovid

#1 (Saudi Arabia OR Saudi Arabia OR Saudi OR Arabia OR Riyadh OR Mecca OR Medina OR Jeddah OR Makkah).ti,ab,kw.

#2 exp Saudi Arabia/

#3 #1 OR #2

#4 (Pediatric Obes$ OR Obes$ OR Overweight OR BMI OR Body Mass Index OR Adiposit$ OR Obesity related diseases).ti,ab,kw.

#5 exp pediatric obesity/

#6 #4 OR #5

#7 (Child$ OR Adoles$ OR Child* OR children* OR Childhood OR Kid OR kids OR Minor$ OR Toddler$ OR Teenager$ OR Young adult$ OR adolescence$).ti,ab,kw.

#8 exp child/ OR exp adolescent/

#9 #7 OR #8

#10 (Prevalen$ OR Epidemiolog$ OR Cross-Sectional Studies OR epidemiology).ti,ab,kw.

#11 exp prevalence/ OR exp epidemiology/ OR exp cross-sectional study/ OR epidemiology.fs.

#12 #10 OR #11

#13 #3 AND #6 AND #9 AND #12

Google Scholar

("Obesity" OR "Overweight" OR "BMI" OR "Body Mass Index" OR "Adiposity" OR "Obesity-related diseases" OR "obesities") AND ("Child*" OR "children*" OR "Childhood" OR "Kid*" OR "Minor" OR "Toddler" OR "Teenager*" OR "Young adult*" OR "adolescent*" OR "adolescency" OR "adolescences") AND ("saudi arabia" OR "Saudi" OR "arabia" OR "Riyadh" OR "Mecca" OR "Medina" OR "Jeddah") AND ("Epidemiology" OR "Prevalence")

Web Of Science

("Obesity*" OR "Overweight*" OR "BMI*" OR "Body Mass Index*" OR "Adiposity*" OR "Obesity-related diseases*" OR "obesities*") AND ("Child*" OR "children*" OR "Childhood*" OR "Kid*" OR "Minor*" OR "Toddler*" OR "Teenager**" OR "Young adult*" OR "adolescent*" OR "adolescency*" OR "adolescences*") AND ("saudi arabia*" OR "Saudi*" OR "arabia*" OR "Riyadh*" OR "Mecca*" OR "Medina*" OR "Jeddah*") AND ("Epidemiology*" OR "Prevalence*")

ProQuest

noft(Obesity* OR Overweight* OR BMI* OR Body Mass Index* OR Adiposity* OR Obesity-related diseases* OR obesities*) AND noft(Child* OR children* OR Childhood* OR Kid* OR Minor* OR Toddler* OR Teenager* OR Young adult* OR adolescent* OR adolescencE* OR adolescences*) AND noft(saudi arabia* OR Saudi* OR arabia* OR Riyadh* OR Mecca* OR Medina* OR Jeddah*) AND noft(Epidemiology* OR Prevalence*) AND pd(19900101-20240320) AND PEER(yes)
